# Transcriptomic identification of miR-205 target genes potentially involved in metastasis and survival of cutaneous malignant melanoma

**DOI:** 10.1038/s41598-020-61637-4

**Published:** 2020-03-16

**Authors:** Beatriz Sánchez-Sendra, Eva Serna, Lara Navarro, Jose F. González-Muñoz, Jesica Portero, Alberto Ramos, Amelia Murgui, Carlos Monteagudo

**Affiliations:** 1 Department of Pathology, Universitat de València, València, Spain; 2 Biomedical Research Institute INCLIVA, València, Spain; 3 Unidad Central de Investigación en Medicina, Facultad de Medicina, Universitat de València, València, Spain; 4 Department of Physiology, Universitat de València, València, Spain; 5 Consortium Hospital General Universitario de València, València, Spain; 6 Department of Biochemistry and Molecular Biology, Universitat de València, València, Spain; 7 Department of Pathology, Hospital Clínico Universitario de Valencia, València, Spain

**Keywords:** Melanoma, Cancer epigenetics

## Abstract

Cutaneous melanoma is an aggressive neoplasm and is responsible for the majority of skin cancer deaths. Several miRNAs are involved in melanoma tumor progression. One of them is miR-205, the loss of which contributes to the development of melanoma metastasis. We evaluated whole-genome mRNA expression profiling associated with different miR-205 expression levels in melanoma cells. Differential expression analysis identified 243 differentially expressed transcripts including inositol polyphosphate 5′-phosphatase-like protein-1 (INPPL1) and BTB/POZ Domain-Containing Protein 3 (BTBD3). INPPL1 and BTBD3 were downregulated when melanoma cells expressed miR-205, indicating that these genes are potential miR-205 targets. Additionally, the target prediction algorithm TargetScan revealed that INPPL1 and BTBD3 genes had predicted target sites of miR-205 in their 3′UTRs and functional analysis demonstrated that these genes were directly linked to miR-205. Interestingly, our clinical data showed that INPPL1 was significantly associated with lymph node metastasis-free survival (LNMFS), distant metastasis-free survival (DMFS) and melanoma specific survival (MSS). This study supports INPPL1 as a miR-205 target gene and, therefore, that the involvement of miR-205 in the metastatic dissemination of malignant melanoma is, at least in part, via INPPL1.

## Introduction

Most skin cancer related deaths are caused by melanoma, a malignant neoplasm with great potential to metastasize that is increasing in incidence and mortality^1^. Epigenetic regulation of gene expression has, as in other cancer types, a significant role in melanoma^2^. These epigenetic events include microRNAs (miRNAs).

miRNAs are short noncoding RNAs with gene regulatory functions. miRNAs modulate gene expression by post-transcriptional repression, as they can direct mRNA cleavage or direct translational repression of their target protein-coding genes^3,4^. Specific miRNAs have been implicated in many biological and pathological processes. miRNA impact on cancer depends on the functional nature of the targeted genes. In this way, miRNAs can produce both oncogenic or tumor suppressive effects by suppressing tumor suppressive mRNAs or oncogenic mRNAs, respectively. With regard to miR-205 target genes in melanoma, Dar *et al*. reported that miR-205 is potentially involved in melanomagenesis through suppression of cell proliferation and induction of senescence via regulation of E2F1 oncoprotein^5^. It has also been suggested that miR-205 controls melanoma cell migration and invasion and that it inhibits the ZEB2 transcription factor^6^.

In particular, some miRNAs are known to be involved in the development and progression of melanoma^7–9^. In this regard, we have recently demonstrated that the intratumoral downregulation of tumor suppressive miR-205 in primary melanomas contributes to the development of distant metastasis, and therefore to a shorter survival. Consequently, miR-205 intratumoral expression levels are useful to predict melanoma metastatic dissemination and patient survival^10^. Our *in vitro* experiments have shown that one of the mechanisms by which miR-205 downregulation may favor metastatic dissemination relies on the interaction of melanoma cells with the extracellular matrix. Thus, it is critical to determine new target genes through which miR-205 mediates its influence on the metastatic process in melanoma.

Whole-genome transcriptome analysis after a specific miRNA overexpression integrated with functional analysis^11^ allows for simultaneous evaluation of a large number of genes (potential targets) to identify the action mechanisms of the miRNA under study and characterize its specific target genes and functions.

In this work, our aim was to clarify the targets and pathways by which miR-205 influences the development of metastasis in human cutaneous melanoma. For this purpose, we conducted a transcriptomic microarray-based profiling study in melanoma cells with and without miR-205 expression in order to analyze differences in gene expression and identify new genes targeted by miR-205 in melanoma.

## Results

### Identification of miR-205 regulated genes by genome-wide gene expression analysis

To identify the genes affected by miR-205, we performed microarray expression analysis of A375 human melanoma miR-205 overexpressing cells and A375 miR-205 negative control cells. Statistical group comparisons of the overexpressing miR-205 cells and controls yielded a total of 243 differentially expressed transcripts derived from an analysis of variance (FDR < 0.05), of which 152 were up-regulated and 91 downregulated (Table 1 Supplementary File). This analysis revealed notable changes in gene expression, suggesting that the upregulation of miR-205 significantly alters the expression profiles of the cell line.

**Figure 1:**
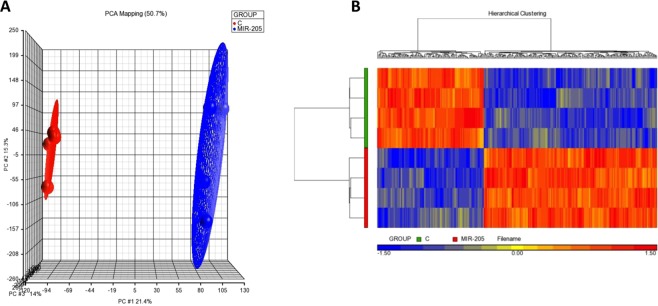
Gene expression patterns between miR-205 transfected melanoma cells (A375) and miR-205 negative controls. (**A**) Three-dimensional unsupervised principal component analysis (PCA) based on the whole genome. PCA shows 4 samples with up-regulated miR-205 (blue) and 4 control samples (red) from A375 cells. Individual samples are plotted based on their respective positions along the three axes. (**B**) Heat-map of the unsupervised hierarchical clustering of up-regulated miR-205 vs control samples showing the 243 differentially expressed genes derived from ANOVA test. Each column represents a gene and each line a sample. Over-expressed genes are represented in red and under-expressed ones in blue. The bars on the left of the panel represent different study groups: the green bar represents control samples (C, n = 4) and the red bar represents up-regulated miR-205 samples (MIR-205, n = 4).

Three-dimensional unsupervised principal component analysis (PCA) based on the whole human genome (more 33,500 coding transcripts and more 11,000 long intergenic non-coding transcripts) of 8 samples from A375 cell line, 4 with up-regulated miR-205 and 4 controls, is represented in Fig. 1A. The PCA revealed that each clustered sample set from the up-regulated miR-205 and control samples were clearly positioned in two different areas from each other.

An unsupervised hierarchical clustering with the 243 significant transcripts derived from ANOVA was performed. Hierarchical clustering ordered the transcripts according to their expression levels, revealing two different clustered gene expression patterns corresponding to up-regulated miR-205 and miR-205 negative controls (Fig. 1B). Microarray gene expression analysis data have been deposited in the ArrayExpress database at EMBL-EBI (www.ebi.ac.uk/arrayexpress) under accession number E-MTAB-8202.

### Functional analysis (Pathway Studio platform)

**Figure 2:**
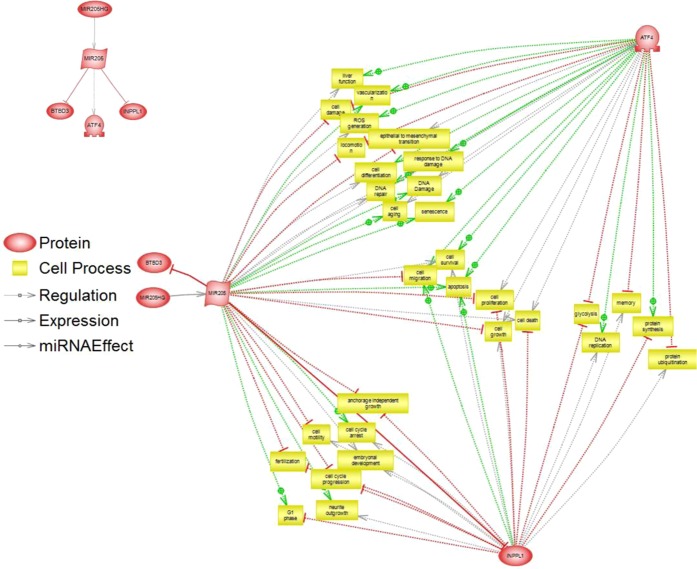
Genes and biological processes associated with miR-205. Association of significant genes directly linked to miR-205 and biological processes associated with miR-205 analyzed by Pathway Studio platform.

**Figure 3:**
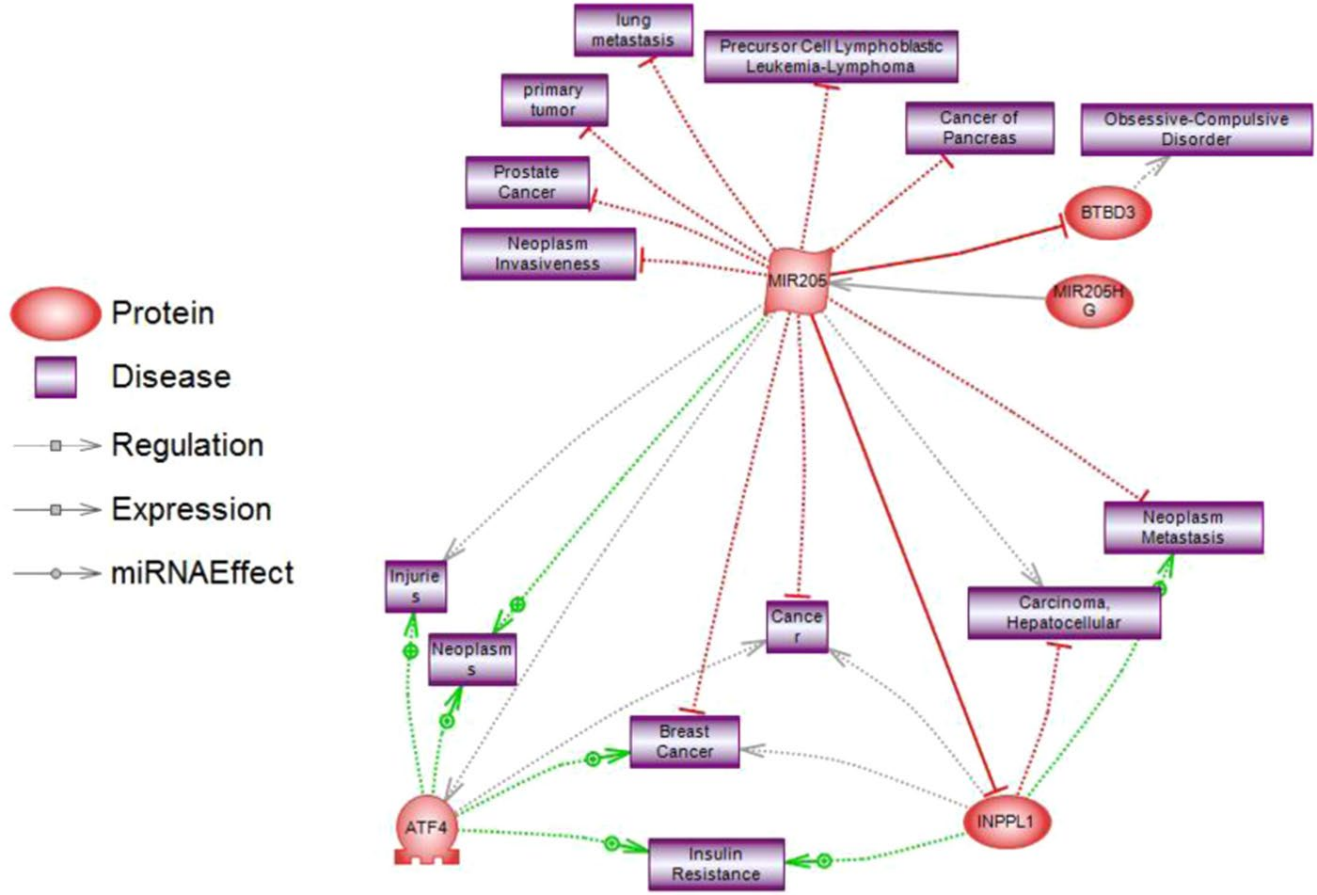
Genes and diseases associated with miR-205. Association of significant genes directly linked to miR-205 and diseases associated with miR-205 analyzed by Pathway Studio platform.

To extract biological meaning from the differentially regulated genes in the miR-205 up-regulated group compared to controls, we investigated the entire list of 243 transcripts through functional annotation analysis using Pathway Studio database (Elsevier). Based on p values after 1 way-ANOVA, these genes represent a number of relevant biological processes such as “Negative regulation of cell proliferation” (p 8.32 × 10^−7^), “Nuclear mRNA splicing” (p 1.08 × 10^−6^), “Mitotic cell cycle” (p 3.17 × 10^−6^) or “Transcription, DNA-dependent” (p 2.63 × 10^−5^), among others. Deepening the biological analysis, we focused on the significant genes having a direct functional relation with miR-205 and, from our list, we identified INPPL1, ATF4 and BTBD3 as significantly miR-205-associated genes. This relationship was filtered using known bibliographic information describing interactions between molecules. Figures 2 and 3 show the biological processes and diseases associated with both miR-205 and the aforementioned identified genes.

As detected from the gene expression data and Pathway Analysis, the most relevant genes among the differentially expressed from both statistical and biological perspectives were miRNA 205-HG (fold change 2.33, p 2.94 × 10^−5^), INPPL1 (fold change −1.63, p 8.66 × 10^−5^), BTBD3 (fold change −1.40, p 1.60 × 10^−4^) and ATF4 (fold change −1.27, p 1.83 × 10^−4^). All three genes were downregulated in miR-205 overexpressing cells compared to the controls. The other genes not selected for further analysis are listed in Table 1 of the Supplementary File.

### Validation by RT-qPCR and target prediction

**Figure 4:**
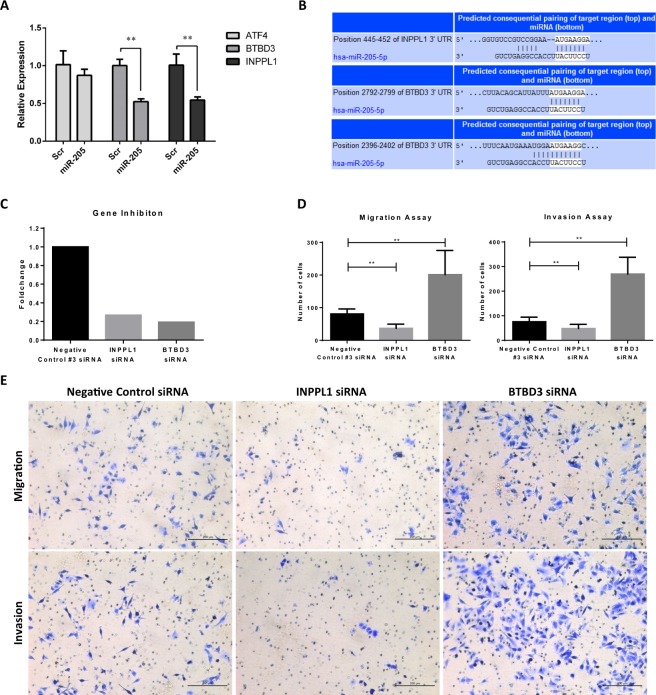
Microarray expression validation, target prediction and functional *in vitro* assays for miR-205 putative target genes. (**A**) Microarray gene expression for ATF4, BTBD3 and INPPL1 was validated by reverse transcription real-time quantitative PCR (RT-qPCR) on the same RNA samples used for the arrays. (**B**) The complementary match between miR-205-5p and the 3′UTR of INPPL1 and BTBD3 is shown as predicted by TargetScan software. (**C**) Validation of the INPPL1 and BTBD3 inhibition using specific siRNAs in A375 cells measured by RT-qPCR. (**D**) Cell counting for the migration and invasion assays on A375 cells for INPPL1 and BTBD3 inhibition compared to A375 negative control cells. (**E**) Representative 10X microscopic images of the migration and invasion functional assays performed on A375 cells. Data represent the mean ± SD of three independent experiments. **p < 0.01.

To confirm the results of the gene expression microarrays, INPPL1, BTBD3 and ATF4 expression was validated by RT-qPCR on the same RNA samples used for the initial arrays. In all samples the expression was lower in miR-205 expressing cells, similar to the results observed in the gene microarray analysis. Such downregulation was significant for INPPL1 and BTBD3 and, although ATF4 was not significant, it showed the same tendency as in the microarray analysis (Fig. 4A). These three putative miR-205 target genes were investigated using TargetScan, which predicted that INPPL1 has one conserved target site of miR-205 in its 3′UTR. In fact, miR-205 is the only miRNA with conserved sites for INPPL1. TargetScan also revealed that BTBD3 mRNA has two, one conserved (position 2792–2799) and one poorly conserved (position 2396–2402), predicted target sites of miR-205 in its 3′UTR (Fig. 4B). However, no matches were found between miR-205 and the 3′UTR of ATF4. The validated and predicted genes, INPPL1 and BTBD3, were inhibited for further functional *in vitro* assays. For clinical samples studies, the downregulated ATF4 gene was also included along with INPPL1 and BTBD3 not to miss any possible clinical associations.

### Functional effect of INPPL1 and BTBD3 inhibition on cellular migration and invasion

Transfection of A375 cells with specific siRNAs caused a significant 73.3% inhibition of INPPL1 (fold change 0.267) and a 80.8% reduction of BTBD3 mRNA levels (fold change 0.192) (Fig. 4C). After this inhibition, we had evaluated the functional effects of INPPL1 and BTBD3 on cellular migration and invasion. As expected, INPPL1 inhibited cells significantly migrated and invaded less than negative control cells (p < 0.001). Nonetheless, BTBD3 inhibited cells showed higher number of cells which migrated and invaded to the lower chamber of the transwell insert (p < 0.001) (Fig. 4D,E).

### Analysis of the expression of miR-205 and its putative target genes (INPPL1, BTBD3 and ATF4) in human primary melanoma specimens

**Figure 5:**
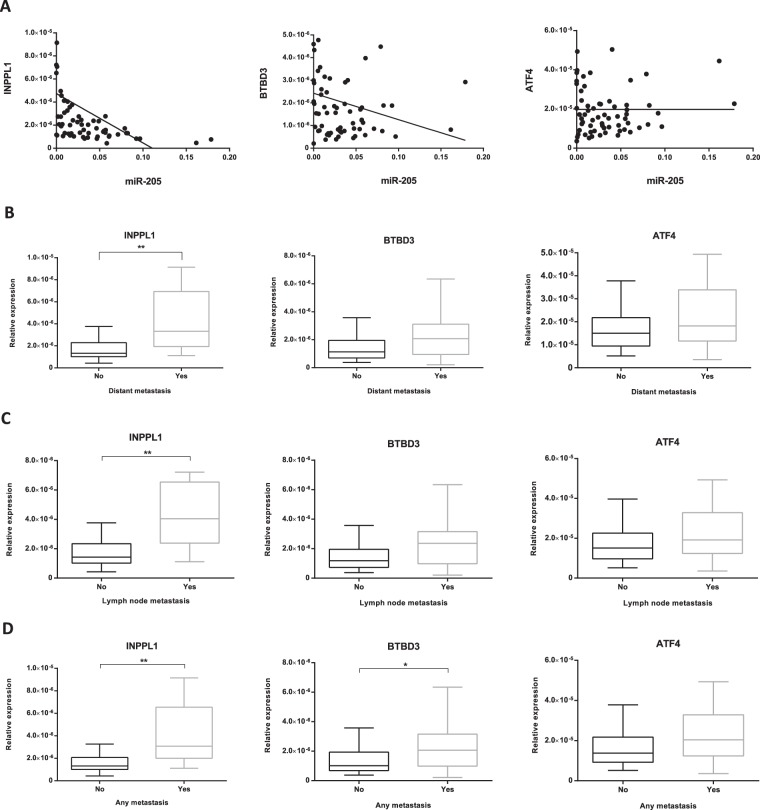
Analysis of miR-205 putative target genes INPPL1, BTBD3 and ATF4 in primary human melanoma tissue samples. (**A**) Correlation of relative expression of INPPL1, BTBD3 and ATF4 with miR-205 relative expression. Association of INPPL1, BTBD3 and ATF4 expression in primary tumors based on the further development of (**B**) distant metastasis, (**C**) regional lymph node metastasis and (**D**) any type of metastasis. *p < 0.05, **p < 0.01.

miR-205, INPPL1, BTBD3 and ATF4 expression was measured by RT-qPCR in human primary melanoma tissue samples. Histological and clinical characteristics of the patients are summarized in Table 1. A significant correlation was found between miR-205 and the expression of its putative target genes INPPL1 and BTBD3 (Spearman r −0.628, p < 0.001; Spearman r −0.295, p 0.017 respectively) whereas no significant correlation was found between miR-205 and ATF4 expression (Spearman r −0.07, p 0.571) (Fig. 5A). We then analyzed the expression profile of the three miR-205 putative target genes in the primary melanoma tumors in relation to their different clinical behavior. Thus, we found INPPL1 was the only gene whose expression was significantly higher in primary melanomas which further developed distant metastasis than in non-metastatic primary melanomas (p 0.002) (Fig. 5B). Likewise, INPPL1 levels were significantly higher in primary tumors which further developed regional lymph node metastasis, LNM, (p 0.002) (Fig. 5C). We also explored possible associations between INPPL1, BTBD3 and ATF4 expression in relation to any type of metastasis. In this regard, not only INPPL1 but both, INPPL1 and BTBD3 expression were significantly higher in primary melanomas that developed any type of metastasis (p < 0.001 and p 0.021, respectively) (Fig. 5D). BTBD3 and ATF4 were found not to be significantly up-regulated with regard to the development of distant or lymph node metastasis (p 0.079 and p 0.119, respectively) (Fig. 5B,C). In all cases, and in full accordance with our previously reported findings, miR-205 expression was significantly lower in primary tumors which further developed distant, lymph node metastasis or any type of metastasis (p < 0.001).

**Table 1:** Histological and clinical characteristics of melanoma patients.

Primary melanomas (N = 68)		
Variable	Number of cases	(%)
**Breslow thickness (mm)**		
≤1 >1	29 39	42.6 57.4
**Ulceration**		
Absent Present	51 17	75.0 25.0
**Mitosis/mm**^2^		
0 ≥1	21 47	30.9 69.1
**Growth phase**		
Radial Vertical	22 46	32.4 67.6
**Location**		
Limbs Trunk Head and neck	27 34 7	39.7 50.0 10.3
**Gender**		
Female Male	45 23	66.2 33.8
Histological type*		
SSM LMM ALM NM	49 5 5 9	72.0 7.4 7.4 13.2
**Age at diagnosis (years)**		
≤65 >65	36 32	52.9 47.1
**In-transit Metastasis**		
Absent Present	58 10	85.3 14.7
**Lymph Node Metastasis**		
Absent Present	53 15	77.9 22.1
**Distant Metastasis**		
Absent Present	48 20	70.6 29.4
**Melanoma Specific Survival**		
Alive Dead	52 16	76.5 23.5
**Follow-up (months)**		
Mean. Range	93	6.6–187.9

^*^Superficial Spreading Melanoma (SSM), Lentigo Maligna Melanoma (LMM),

Acral lentiginous Melanoma (ALM) and Nodular Melanoma (NM).

**Figure 6:**
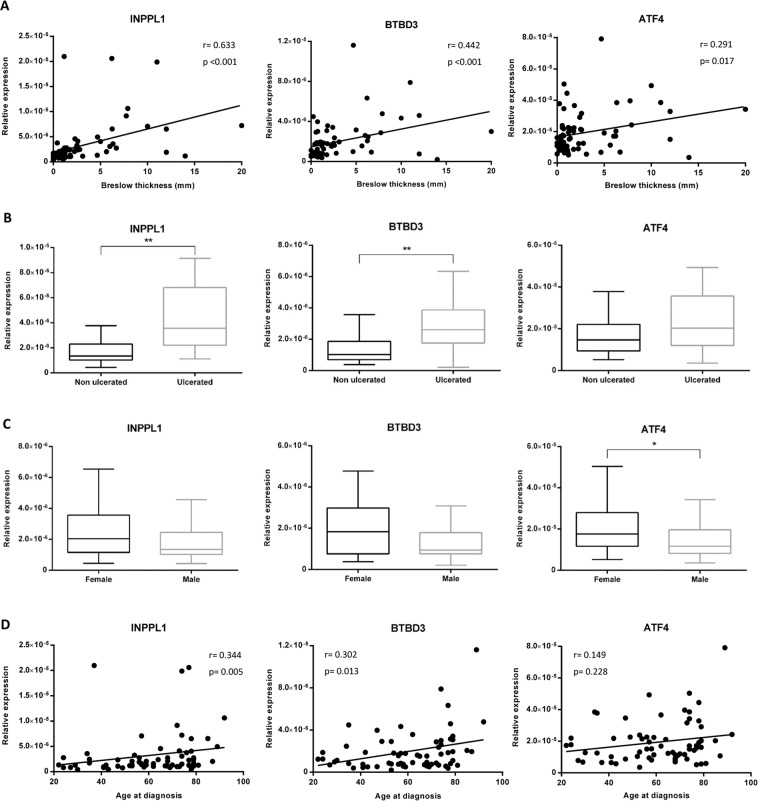
INPPL1, BTBD3 and ATF4 expression in relation to Breslow thickness, ulceration and patient gender and age at diagnosis. Association between the relative expression of INPPL1, BTBD3 and ATF4 with (**A**) Breslow thickness and (**B**) ulceration status of the primary melanomas; and with (**C**) gender and (**D**) age at the moment of diagnosis of the patients. *p < 0.05, **p < 0.01.

Additionally, we investigated the association of INPPL1, BTBD3 and ATF4 expression with histological tumor parameters such as Breslow thickness and ulceration as well as with patient gender and age at diagnosis. These results showed a significant direct correlation between INPPL1, BTBD3 and ATF4 expression and Breslow thickness (Spearman r 0.633, p < 0.001; Spearman r 0.442, p < 0.001 and Spearman r 0.291, p 0.017 respectively) (Fig. 6A). INPPL1 and BTBD3 expression was significantly higher in ulcerated than in non-ulcerated primary melanomas (p < 0.001 in both cases). No significant relation with ulceration status was found for ATF4 expression (Fig. 6B). Regarding patient gender (females n = 45, males n = 23), we observed that INPPL1 and BTBD3 had consistent expression between genders since males showed lower levels of ATF4 than females (p 0.014) (Fig. 6C). With respect to age at diagnosis, the results revealed that INPPL1 and BTBD3 expression directly correlated with age (Spearman r 0.344, p 0.005; Spearman r 0.302, p 0.013 respectively) whilst ATF4 expression did not show any association with age (Fig. 6D).

### Association of INPPL1, BTBD3 and ATF4 with lymph node metastasis-free survival, distant metastasis-free survival and melanoma specific survival

**Figure 7:**
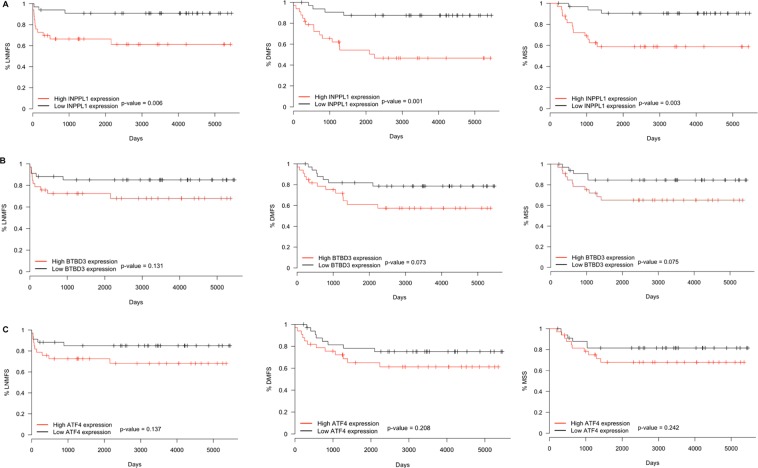
Kaplan-Meier curves for lymph node metastasis-free survival (LNMFS), distant metastasis-free survival (DMFS) and melanoma specific survival (MSS) of melanoma patients. Impact of (**A**) INPPL1, (**B**) BTBD3 and (**C**) ATF4 expression on the LNMFS, DMFS and MSS of patients.

Survival analysis (Kaplan-Meier curves) showed that INPPL1 was significantly associated with lymph node metastasis-free survival (LNMFS), distant metastasis-free survival (DMFS) and melanoma specific survival (MSS). All three (LNMFS, DMFS and MSS) were significantly shorter in patients with primary melanomas showing INPPL1 levels above the median (Fig. 7A). BTBD3 and ATF4 gene expression did not show significant correlations with LNMFS, DMFS or MSS (Fig. 7B,C).

In order to ascertain the most appropriate model to predict lymph node metastases and distant metastases, logistic and Cox regression analyses were performed. Variables miR-205, INPPL1, BTBD3 and ATF4 were included in the model. By logistic regression, the most appropriate predictive model for any of the previous clinical outcomes found miR-205 as the only explanatory variable in such a way that the more miR-205, the lower probability of developing any metastatic event and the higher probability of survival. By Cox regression, similar to the logistic regression results, the most suitable predictive model also showed miR-205 as the sole explanatory variable, meaning that in the presence of miR-205 variable, the target genes do not provide additional significant information and, as the miR-205 hazard ratio indicates, the higher miR-205, the lower risk of metastasis and, therefore, longer patient survival (Table 2).

**Table 2:** Multivariate stepwise Cox regression analysis for LNMFS and DMFS.

	HR (Exp(B))	95% CI	p value
**LNMFS**			
**Initial step** miR-205 ATF4 BTBD3 INPPL1	0.000 1.016 0.842 1.032	0.000–0.000 0.960–1.075 0.541–1.311 0.919–1.159	0.008* 0.581 0.446 0.595
**Final step** miR-205	0.000	0.000–0.000	0.006*
**DMFS**			
**Initial step** miR-205 ATF4 BTBD3 INPPL1	0.000 1.010 0.914 1.057	0.000–0.000 0.964–1.058 0.636–1.311 0.965–1.156	0.001* 0.682 0.624 0.232
**Final step** miR-205	0.000	0.000–0.000	0.000*

Variables included in the prediction model: miR-205, INPPL1, BTBD3 and ATF4 expression.

## Discussion

Dysregulation of miR-205 is one of the epigenetics events involved in the progression of melanoma. miR-205 regulates multiple cellular functions, including proliferation, migration and invasion, and is commonly altered in cancer^10^. Advances in the identification of the target genes for miR-205 may significantly contribute to delineating and understanding the molecular pathways involved in the metastatic dissemination of malignant melanoma when miR-205 expression is downregulated. This knowledge could provide new potential therapeutic tools to manipulate these target genes in order to block the metastatic capacity of melanoma cells.

We have previously reported that miR-205 expression directly correlates with distant metastasis-free survival and inversely with melanoma specific mortality^10^. Among the 243 differentially expressed transcripts found in the transcriptomic study, our functional and target prediction analyses indicated two new putative miR-205 target genes: INPPL1 and BTBD3.

Our *in vitro* inhibition results support INPPL1 as the main responsible for melanocytic cell migration and invasion and, therefore, the miR-205-mediated suppression of melanoma progression would be due, at least in part, to the reduced expression of INPPL1. In contrast, BTBD3 inhibition did not imply a reduction of the invasion ability of melanoma cells.

In clinical samples, results demonstrate that INPPL1 and BTBD3 expression inversely correlates with miR-205 what again indicates that INPPL1 and BTBD3 may be potential miR-205 target genes. At the same time, INPPL1 and BTBD3 levels directly correlate with Breslow thickness and are higher in ulcerated than in non-ulcerated tumors. Besides, INPPL1 and BTBD3 expression was independent of patient gender and exhibited a direct correlation with age. More importantly, INPPL1 correlates with the development of distant and lymph node metastasis and, both, INPPL1 and BTBD3, with the development of any type of metastasis. When the time to the event is taken into consideration, our findings demonstrate an inverse correlation of INPPL1 with lymph node and distant metastasis-free survivals and a direct correlation with melanoma specific mortality. These findings suggest a potential influence for the most part of INPPL1 and may also for BTBD3 on promoting melanoma progression through a miR-205-downregulation mediated mechanism, which is consistent with our previous results^10^.

Inositol polyphosphate 5′-phosphatase-like protein-1 (INPPL1), also known as Src homology 2 (SH2) domain-containing inositol 5′-phosphatase 2 (SHIP2), dephosphorylates phosphatidylinositol 3,4,5-trisphosphates (PIP3) generated by PI3K. SHIP2 mRNA is ubiquitously expressed and its protein levels are thought to be regulated at post-transcriptional level^12^. SHIP2 typically localizes in the cytosol, but once stimulated, is rapidly translocated to the plasma membrane where it dephosphorylates its substrates and mediates protein-protein interactions^13^. SHIP2 is implicated in various cellular processes and diverse diseases. It positively regulates cellular adhesion, lamellipodia formation and cell spreading^14^, and remodels actin cytoskeleton organization of HeLa cells^15^. SHIP2 function has been shown to be important in the molecular pathogenesis of diverse diseases such as inflammation, diabetes, obesity, neurodegenerative disorders, atherosclerosis and cancer^16^.

Concerning the involvement of SHIP2 in diabetes, Langlet *et al*. showed that miR-205-5p overexpression increased AKT phosphorylation and decreased SHIP2 at mRNA and protein level in primary hepatocytes which indicates that miR-205-5p enhances insulin signaling *in vitro*^17^. Regarding the role of SHIP2 in cancer, it has been reported to have both tumor-suppressive and pro-oncogenic effects. The tumor-suppressive role of SHIP2 was found by Taylor *et al*. who observed that exogenous overexpression of SHIP2 negatively regulated AKT signalling pathway and repressed cell cycle progression in U87-MG glioblastoma cells to the same extent as PTEN^18^. SHIP2 overexpression has also been reported to inhibit cell cycle progression in K562 leukemia cells^19^. In contrast, SHIP2 has also been shown to act as an oncogene. SHIP2 protein levels were observed to be higher in breast cancer samples compared to non-cancerous breast tissues, and also found to be higher in breast cancer cell lines compared to normal breast lines^20^. Functionally, Prasad *et al*. showed that silencing of endogenous SHIP2 in MDA-231 cells leads to a reduction in proliferation and delays tumor formation in nude mice^20^. The same authors found in an MDA-231 breast cancer cell line that SHIP2 promotes cell migration and this effect is associated with sustained EGFR-AKT signaling and increased expression of chemokine receptor CXCR4^21^. SHIP2 may also act as an oncogene contributing to the malignant potential of colorectal cancer by regulating PKB activation^22^. In stratified squamous epithelia, Yu *et al*. described that SHIP2 protein levels are regulated post-transcriptionally by miR-205 and miR-184 (which is a suppressor of miR-205)^23^ and that miR-205 promotes keratinocyte migration via lipid phosphatase SHIP2^24^. In the latter study, carried out in a keratinocyte model, miR-205 directly suppressed the expression of SHIP2, leading to the increased activation of AKT, followed by the promotion of migration and the repression of adhesion. This suggests that miR-205 may have multiple roles and that its function may be tissue specific.

At present, there are limited studies on BTB/POZ Domain-Containing Protein 3 (BTBD3) involvement in cancer. Xiao *et al*. reported that the BTBD3 gene was up-regulated in hepatocellular carcinoma (HCC) compared with normal tissues^25^. Based on their *in vitro* inhibition experiments, these same authors suggested that BTBD3 gene promotes metastatic dissemination of HCC^25^. Zhang *et al*. exploring the function of hsa-let-7i in colorectal cancer cell lines, predicted eight genes, including BTBD3, as potential hsa-let-7i targets^26^.

To the best of our knowledge, there is no prior information about the involvement of INPPL1 and BTBD3 in melanoma. Thus, our study reports for the first time their implication in melanoma progression as miR-205 targets. *In vitro* inhibition of endogenous INPPL1 led to a reduction in cell migration and invasion supporting the potential pro-oncogenic role of INPPL1 in melanoma, but BTBD3 inhibition did not show the same expected effect. These seemingly contradictory results can be attributed to a number of reasons including off-target effects of the antisense BTBD3 reagents. Besides this, cellular migration and invasion are widely regulated processes affected by multiple regulators. Somehow, functional redundancy, whereby the loss of one gene may be compensated by other genes performing the same function, could explain the induction of cell migration and invasion in response to BTBD3 inhibition and, in addition, *in vitro* results are less reliable than results obtained from clinical samples since *in vitro* conditions do not replicate the real and complex human *in vivo* environment.

Among INPPL1 and BTBD3, INPPL1 was found to be the most clinically relevant gene that seems to be mediator of miR-205 suppression of melanoma progression. Similar to miR-205, INPPL1 has an important prognostic value, but interestingly it does not contribute additional prognostic information to miR-205, supporting the hypothesis that miR-205 influences melanoma clinical aggressiveness, and therefore patient survival through the inhibition of, at least, its herein newly described pro-oncogenic INPPL1 target gene. In summary, our results support the value of INPPL1 miR-205-regulated gene as a prognostic biomarker of human melanoma, which may be helpful when miRNA expression cannot be determined. A limitation of this study is that, though global clinical follow-up comprises 187.9 months, for a small subset of cases the clinical follow-up is shorter. Molecular mechanisms by which INPPL1 and BTBD3 affect melanoma progression require further investigation as well as their potential therapeutic value in order to block the metastatic dissemination of cutaneous malignant melanoma.

## Methods

This work has partially followed the indications on methodology previously described by Sánchez-Sendra *et al*.^10^.

### Melanoma specimens and cell culture

The study included 68 melanoma primary tumors from patients with cutaneous melanoma. All tumor specimens were received at the Department of Anatomic Pathology, Hospital Clínico Universitario, Valencia, between 2002 and 2014. Clinical follow-up ranged from 6.6 to 187.9 months (mean 93, median 97.4 months). All tumors were classified according to the 2017 American Joint Committee on Cancer (AJCC) staging system. Our study was approved by the Ethical and Scientific Committees of the Hospital Clínico Universitario of Valencia and all research was performed in accordance with relevant guidelines and regulations. Written informed consent was obtained from each patient. The melanoma cell line used for the transcriptomic study and for migration and invasion assays was the A375 melanoma cell line. A375 cells were incubated in DMEM medium with glucose and L-glutamine supplemented with 10% fetal bovine serum, penicillin and streptomycin (Gibco) and maintained in a humidified incubator (5% CO_2_) at 37 °C. Cells were routinely tested using MycoAlert™ mycoplasma detection kit (Lonza).

### RNA extraction and RNA integrity

Total RNA, was extracted from melanoma tissues and cultured cells using the mirVana miRNA isolation kit (Ambion, Austin, TX, USA) according to the manufacturer’s instructions. RNA concentration and quality from human melanoma tissues were determined on a NanoDrop One spectrophotometer (Thermo Fisher Scientific). RNA quality and integrity from cultured cells was checked by capillary electrophoresis using the 2100 Bioanalyzer (Agilent). Hybridization to whole transcript expression microarrays was performed only if the RNA integrity number (RIN) was ≥7.

### Gene expression microarray analysis

For whole-genome mRNA expression profiling, four biologically independent replicates from A375 human melanoma miR-205 overexpressing cells (miR-205) and A375 miR-negative control cells (Scr) were analyzed using GeneChip Human Gene 2.0 ST Arrays (Affymetrix®, Santa Clara, CA, USA) following the manufacturer’s protocol. Microarray experiments were performed at the Central Research Unit of Medicine (University of Valencia). Human A375 melanoma miR-205 overexpressing cells and control cells used in these experiments were obtained as previously described^10^.

Files were captured using an Affymetrix^®^ GeneChip Scanner 3000 7G at 570 nm wavelength excitation. Data (.CEL files) were used to analyze significant changes in gene expression profiles and were statistically filtered using software Partek Genomic Suite 6.6 (Partek Inc., St. Louis, MO, USA). Input files were normalized with the robust multiple-array average (RMA) algorithm for gene array on core meta probesets. Next, a one-way ANOVA was performed with the Partek Genomics Suite across all samples. Statistically significant genes between up-regulated miR-205 cells and controls were identified using a model analysis of variance of Fold Discovery Rate (FDR) < 0.05. Significant genes derived from ANOVA were analyzed by Principal Components Analysis (PCA) to determine the significant global transcriptome differences and were ordered according to their expression levels in an unsupervised hierarchical clustering. Finally, the selected differentially expressed genes were imported into Pathway Studio version 10 (Ariadne Genomics^®^ software, Elsevier^®^ Inc, Rockville, MD, USA) to classify biological processes and associations between genes. The predicted target genes and their miRNA binding site seed regions were investigated using TargetScan (release 5.1, http://www.targetscan.org/).

### miRNA quantification by RT-qPCR

miRNA quantification data from human melanoma specimens to evaluate miR-205 expression in clinical samples was taken from our previous study^10^. A few clinical samples were new and then analyzed for the first time for this work.

### mRNA quantification by RT-qPCR

From human melanoma samples, mRNA quantification was performed to evaluate INPPL1, BTBD3 and ATF4 mRNA expression in clinical samples, and was also done from cultured cells to validate microarray results and to validate gene expression after INPPL1 and BTBD3 inhibition experiments. In all cases, relative quantification of INPPL1, BTBD3 and ATF4 mRNAs was performed by reverse transcription real-time quantitative PCR (RT-qPCR). Reverse transcription (RT) was performed using the High Capacity cDNA Reverse Transcription Kit adding RNase inhibitor (Lifetechnologies). In 50-µl reactions, 150 ng of total RNA was converted to cDNA. Briefly, 1 µl of cDNA was used in 10-μl qPCR reactions by using TaqMan Gene Expression Master Mix and TaqMan Gene Expression Assays for the target genes INPPL1 (Hs00155533_m1), BTBD3 (Hs01082298_m1) and ATF4 (Hs00909569_g1) and the endogenous reference gene 18S rRNA (Hs99999901_s1) (Lifetechnologies). All reactions were performed in triplicate in 384-well plates on a 7900 HT Fast real-time PCR system (Lifetechnologies). Both, RT and qPCR negative controls were included for each assay. For the analysis, the comparative Ct method was employed and the results were evaluated using Expression Suite software (Lifetechnologies).

### INPPL1 and BTBD3 inhibition and cellular migration and invasion assays

Silencer select specific siRNAs were used to inhibit INPPL1 (ID s7463 – validated, Ambion) and BTBD3 (ID s22631 – pre-designed, Ambion) in A375 melanoma human cells. Silencer negative Control #3 siRNA (AM4615, Ambion) was used as the negative control that does not target any gene product. Briefly, 300.000 A375 melanoma cells were seeded in 6-well plates. 24 hours after seeding, cells were transfected with 6 nM of each of the siRNAs using Lipofectamine RNAiMAX (Invitrogen) following the forward transfection method indicated by manufacturer’s protocol. Cells were maintained in the medium used for transfection for 24 hours and then they were collected for RNA extraction and for migration and invasion assays seeding. Migration and invasion assays were performed as previously described^10^. Cells that migrated or invaded to the downside of the Transwell insert after 48 hours were photographed and scored by imaging. Experiments were done in triplicate.

### Statistical analysis

Statistical analysis was performed using R software package and GraphPad Prism V.6.01 (GraphPad Software, Inc.). The correlation between miR-205 and INPPL1, BTBD3 and ATF4 mRNA expression was assessed by Spearman correlation. The association between mRNA expression and the development of metastasis as a categorical variable was analyzed using the bivariant Mann-Whitney U test. Survival analysis was performed using Log-rank test (Kaplan-Meier curves). Logistic and Cox regressions were studied using stepwise selection to identify independent predictors of clinical outcome. For all statistical analysis, a p value of less than 0.05 was considered significant.

## Supplementary information

Supplementary Information.

## Data Availability

The authors will make materials, data and associated protocols promptly available to readers upon publication in Scientific Reports without undue qualifications in materials transfer agreements.
