# Supplementary material for: Transcriptomic identification of miR-205 target genes potentially involved in metastasis and survival of cutaneous malignant melanoma
Source: Sci Rep. 2020 Mar 16;10:4771. doi: 10.1038/s41598-020-61637-4 (PMC7075905; doi:10.1038/s41598-020-61637-4)
Supplement: Supplementary file 1 — Supplementary Information. [file 41598_2020_61637_MOESM1_ESM.pdf]

Transcriptomic identification of miR-205 target genes potentially involved in metastasis and survival of cutaneous malignant melanoma.

Authors:

Beatriz Sánchez-Sendra[1,2], Eva Serna[3,4], Lara Navarro[1,5], Jose F. González-Muñoz[2], Jesica Portero[3], Alberto Ramos[2], Amelia Murgui[6], Carlos Monteagudo.[1,2,7]*

Affilitiations:

1. Department of Pathology, Universitat de València, Spain.
2. Biomedical Research Institute INCLIVA. València, Spain.
3. Unidad Central de Investigación en Medicina, Facultad de Medicina, Universitat de València, Spain.
4. Department of Physiology, Universitat de València, Spain.
5. Consortium Hospital General Universitario de València, Spain.
6. Department of Biochemistry and Molecular Biology, Universitat de València, Spain.
7. Department of Pathology, Hospital Clínico Universitario de Valencia. València, Spain.

Supplementary Results

Transcript ID	Gene Symbol	RefSeq	p value (MIR-205 vs C)	Fold Change (MIR-205 vs C)
16761012	A2M	NM_000014	6.27E-05	-2.50
16981444	AADAT	NM_001286683	5.15E-05	1.32
16865112	AC008440.10	OTTHUMT00000313724	1.73E-05	1.32
16755602	ACTR6	ENST00000549977	1.41E-04	-1.08
16804478	AEN	XM_005254967	1.34E-04	1.21
16668439	AHCYL1	NM_001242673	7.29E-05	-1.23
16769481	ALDH1L2	NM_001034173	2.31E-04	-2.14
17076634	ANK1	NM_000037	4.17E-05	-1.24
16714618	ANK3	NM_001149	1.15E-05	-1.27
16979638	ANKRD50	NM_020337	1.13E-04	1.20
17084363	ANXA2P2	NR_003573	1.19E-05	1.18
16988174	AP3S1	ENST00000316788	1.52E-06	1.58
16967853	AREG	NM_001657	6.16E-05	1.42
16705199	ARID5B	NM_001244638	1.23E-04	-1.31
17048859	ARPC1A	NM_001190996	2.30E-04	1.15
16683317	ASAP3	NM_001143778	1.45E-04	-1.61
16832852	ATAD5	NM_024857	1.84E-04	1.27
16930299	ATF4	NM_001675	1.83E-04	-1.27
17009853	BAG2	NM_004282	1.23E-04	1.42
16725783	BEST1	ENST00000524877	2.26E-05	-1.79
16705089	BICC1	NM_001080512	5.46E-07	-1.92
16825468	BOLA2	NM_001031827	1.62E-04	1.22
16825683	BOLA2	NM_001031827	1.62E-04	1.22
16899086	BOLA3	NM_212552	1.51E-04	1.28
16847432	BRIP1	NM_032043	1.14E-04	1.33
16911463	BTBD3	NM_014962	1.60E-04	-1.40
16680935	C1orf174	NM_207356	1.62E-04	1.38
16781002	CARS2	NM_024537	7.16E-05	1.29
16800374	CASC4	NM_138423	1.42E-04	-1.34
17052776	CASP2	NM_001224	1.80E-04	1.35
16926111	CBS	NM_001178008	1.07E-04	-1.58
16852966	CCDC102B	NM_001093729	2.50E-04	-1.35
16958083	CCDC58	NM_001017928	8.74E-05	1.20
16953279	CDC25A	NM_001789	1.10E-04	1.61
16877762	CENPO	ENST00000380834	5.79E-05	1.29
16978976	CFI	NM_000204	1.64E-05	-1.35
16733104	CHEK1	NM_001114121	3.66E-05	1.17
16815828	CIITA	NM_000246	2.09E-04	-1.22
17032027	CLIC1	NM_001288	2.46E-04	1.13

17036745	CLIC1	NM_001288	1.29E-04	1.13
17039540	CLIC1	NM_001288	1.29E-04	1.13
17042023	CLIC1	NM_001288	1.29E-04	1.13
17034497	CLIC1	NM_001288	8.81E-05	1.15
16827123	CMTM4	NM_178818	1.68E-06	-1.71
17051553	CPA4	NM_001163446	1.38E-05	3.62
16984182	CTD-2116N24.1	OTTHUMT00000367332	1.95E-04	-1.38
17109367	CTPS2	NM_001144002	6.56E-06	-1.22
16940705	DAG1	NM_001165928	2.03E-04	1.20
16825794	DCTPP1	NM_024096	3.26E-06	1.50
16869624	DDX39A	NM_005804	2.81E-05	1.35
16927174	DGCR8	NM_001190326	3.15E-06	1.63
16687618	DHCR24	NM_014762	1.22E-04	1.22
16974873	DHX15	NM_001358	8.70E-07	1.22
16909081	DOCK10	NM_001290263	2.07E-04	1.30
16867378	DPP9	NM_139159	7.80E-05	1.31
17075973	DUSP4	NM_057158	9.14E-05	1.37
17121122	DUXAP10	NR_110526	1.18E-04	1.27
17111725	EDA2R	NM_001199687	2.56E-06	1.26
16681370	ENO1	NM_001201483	1.95E-04	1.24
17080516	ENPP2	NM_001040092	1.30E-04	-2.11
16682098	EPHA2	XM_005245751	1.37E-04	1.30
17000305	FAM13B	NM_016603	4.36E-05	1.42
16937505	FANCD2	NM_001018115	1.62E-04	1.23
16917061	FERMT1	NM_017671	1.19E-05	1.45
17124708	FKBP9	NM_001284341	1.34E-04	-1.29
17013014	FLJ46906	NR_033896	6.85E-06	-1.90
16863287	FOSB	NM_001114171	4.02E-06	-1.69
16957884	FSTL1	NM_007085	7.53E-05	-1.29
16884062	GCC2	NM_181453	2.05E-04	-1.55
16912192	GINS1	NM_021067	1.57E-04	1.33
17112769	GLA	NM_000169	1.44E-04	1.27
17092268	GLDC	NM_000170	1.83E-04	-1.46
17005396	GMNN	NM_001251989	1.30E-04	1.60
16686510	GPBP1L1	NM_021639	1.10E-04	1.11
16860709	GPI	NM_000175	9.04E-07	1.23
17019778	GPR110	ENST00000419892	1.31E-05	2.57
16847321	HEATR6	NM_022070	9.12E-05	-1.15
16707695	HELLS	NM_001289067	1.38E-04	1.44
17012304	HEY2	NM_012259	2.19E-04	-2.23
17016366	HIST1H2AB	NM_003513	2.09E-05	1.53
16685935	HIVEP3	NM_001127714	1.03E-04	1.20
17032519	HLA-DOA	NM_002119	1.70E-04	-1.85
17034837	HLA-DOA	NM_002119	1.70E-04	-1.85

17018025	HLA-DOA	NM_002119	1.68E-04	-1.80
17037317	HLA-DOA	NM_002119	1.68E-04	-1.80
17042534	HLA-DOA	ENST00000426685	1.48E-05	-1.78
17040023	HLA-DOA	ENST00000383226	1.11E-04	-1.77
17033704	HLA-DPB1	NM_002121	1.68E-04	-1.33
17038687	HLA-DPB1	NM_002121	2.49E-04	-1.32
17028326	HLA-DQA1	ENST00000383251	1.05E-04	-1.51
17041240	HLA-DQA1	ENST00000418023	3.35E-05	-1.49
17038609	HLA-DQA1	ENST00000383251	6.65E-05	-1.48
17031153	HLA-DQA1	ENST00000461508	1.10E-06	-1.46
17033617	HLA-DQA1	ENST00000474698	7.00E-06	-1.44
17028345	HLA-DQA2	NM_020056	7.07E-05	-1.42
17031178	HLA-DQA2	NM_020056	5.36E-05	-1.39
17033646	HLA-DQA2	NM_020056	1.33E-05	-1.38
17035918	HLA-DQA2	ENST00000241802	1.74E-04	-1.37
17038629	HLA-DQA2	ENST00000241802	2.48E-05	-1.36
17034749	HLA-DQB2	ENST00000323109	8.74E-05	-1.29
17039935	HLA-DQB2	ENST00000323109	8.74E-05	-1.29
17095318	HNRNPK	NM_002140	1.69E-05	1.10
16777896	HSPH1	NM_001286503	3.09E-05	1.29
16720085	IFITM1	NM_003641	2.00E-04	-1.47
16728652	INPPL1	NM_001567	8.66E-05	-1.63
16694716	ISG20L2	NM_030980	1.23E-05	1.36
16984689	ITGA2	NM_002203	8.76E-07	1.74
16887702	ITGA6	NM_000210	1.76E-06	1.34
17111484	ITIH6	NM_198510	1.12E-04	-1.94
16938271	KAT2B	NM_003884	2.31E-06	-1.59
17094761	KLF9	NM_001206	1.65E-04	-1.58
16764791	KRT80	NM_182507	1.28E-04	1.80
16802461	LINC00277	NR_026949	1.33E-04	1.29
17121126	LINC01296	NR_122111	1.47E-05	1.29
16671914	LMNA	NM_001257374	1.27E-05	1.32
16760981	M6PR	ENST00000541507	1.91E-04	1.14
16702571	MCM10	NM_018518	2.17E-04	1.48
17068782	MCM4	NM_005914	1.18E-05	1.27
16988728	MEGF10	NM_001256545	2.90E-06	3.28
17125550	MFN1	NM_033540	1.54E-04	-1.12
16676954	MIR205HG	NM_001104548	2.94E-05	2.33
17118868	MIR614	NR_030345	3.72E-05	1.59
17101677	MOSPD2	NM_152581	1.80E-04	-1.28
16991447	MRPL22	NM_001014990	2.01E-04	1.06
16861907	MRPS12	NM_021107	9.49E-05	1.28
16951097	MRPS25	NM_022497	3.63E-05	1.19
16739317	MTA2	NM_004739	9.50E-05	1.20

17083975	MTAP	NM_002451	2.53E-04	-1.23
16720601	MUC5B	NM_002458	2.38E-04	1.28
16716590	MYOF	NM_013451	9.43E-05	1.24
16856910	NCLN	NM_020170	9.72E-05	1.22
16919769	NCOA5	NM_020967	4.38E-05	1.43
16928824	NF2	NM_000268	7.90E-06	1.30
16920315	NFATC2	NM_001136021	8.13E-05	-1.32
17003479	NHP2	NM_001034833	1.85E-05	1.26
16660785	NIPAL3	XM_005245948	1.36E-04	-1.36
16836131	NME1	NM_000269	4.58E-05	1.19
16910825	NOP56	NR_027700	1.96E-04	1.32
16713187	NRP1	NM_001024628	1.44E-06	1.67
17090444	NUP214	NM_005085	1.67E-04	1.12
16818600	ORC6	NM_014321	1.09E-04	1.48
16790032	OSGEP	NM_017807	1.30E-04	1.50
16732850	PANX3	NM_052959	1.70E-04	-1.40
16979339	PDE5A	NM_001083	1.74E-04	1.94
17070926	PDP1	NM_001161779	1.70E-04	1.43
16960442	PFN2	NM_053024	2.27E-05	1.26
16759604	PGAM5	NM_001170543	2.71E-05	1.24
16737669	PHF21A	NM_001101802	9.21E-05	-1.40
16890602	PKI55	NR_037701	9.26E-05	1.23
16811372	PKM	NM_001206796	3.45E-05	1.23
17019728	PLA2G7	NM_001168357	2.40E-04	1.44
17062985	PODXL	NM_001018111	1.07E-04	-1.42
16830488	POLR2A	NM_000937	4.50E-05	1.35
16725935	POLR2G	ENST00000525455	2.29E-04	1.32
16895848	PPM1G	NM_177983	8.64E-07	1.13
16708533	PPRC1	NM_001288727	2.22E-04	1.21
16820414	PRMT7	NM_001184824	1.46E-04	1.28
17123310	PROS1	NM_000313	5.00E-05	-1.37
17123308	PROSP	ENST00000474651	4.67E-05	-1.42
16920910	PSMA7	NM_002792	9.46E-05	1.20
16762661	PTHLH	NM_002820	1.31E-04	1.46
17057378	PURB	NM_033224	2.70E-05	1.33
17092252	RANBP6	NM_001243202	1.53E-04	1.26
17098103	RBM18	NM_033117	7.34E-05	1.04
16669830	RBM8A	NM_005105	8.07E-05	1.29
16938527	RBMS3	NM_001003792	9.95E-05	-1.66
17070013	RDH10	NM_172037	6.48E-05	1.52
16813062	RLBP1	NM_000326	3.31E-05	1.86
16829972	RNF167	NM_015528	5.50E-05	1.22
16842991	RNU6-711P	ENST00000517255	7.67E-05	-1.23
16783439	RP11-317N8.3	OTTHUMT00000409613	4.17E-05	1.19

17121244	RP11-492D6.3	OTTHUMT00000414653	1.92E-04	-1.35
17094042	RP11-613M10.9	OTTHUMT00000401856	1.22E-04	-1.24
17110369	RP6-99M1.2	OTTHUMT00000467765	4.77E-05	2.16
16822919	RPS2	ENST00000531065	6.16E-05	1.49
17092737	RPS6	NM_001010	1.26E-04	1.09
16890915	RQCD1	NM_001271634	1.60E-05	1.36
17069545	RRS1	NM_015169	1.65E-04	1.20
16861734	RYR1	NM_000540	1.16E-05	-1.15
17087498	SEC61B	NM_006808	1.33E-04	1.22
16934012	SELM	NM_080430	1.02E-04	-1.21
16811638	SEMA7A	NM_001146029	1.20E-05	-1.50
16787902	SERPINA3	NM_001085	1.10E-04	-1.42
16906000	SESTD1	NM_178123	8.54E-05	-1.40
16695769	SH2D1B	ENST00000493550	9.72E-05	2.56
16826160	SHCBP1	NM_024745	1.46E-04	1.26
17056072	SKAP2	NM_003930	1.20E-04	-1.31
16984032	SKP2	NM_001243120	7.14E-05	1.41
17059974	SLC25A13	NM_001160210	7.54E-05	1.14
16846476	SLC35B1	ENST00000240333	2.31E-04	1.20
16732985	SLC37A2	NM_001145290	1.89E-04	1.47
16996605	SMIM15	NM_001048249	1.33E-04	1.12
16914741	SNORD12C	ENST00000386307	1.66E-04	1.56
16696533	SNORD75	NR_003941	1.76E-04	1.76
16911605	SNRPB2	NM_003092	8.01E-06	-1.22
16868838	SPC24	NM_182513	1.41E-04	1.40
17111594	SPIN4	NM_001012968	8.27E-06	1.31
17110071	SRPX	NM_001170750	6.19E-05	-1.17
16876310	SRSF10	NM_001191005	4.92E-05	1.28
17075553	STC1	NM_003155	3.59E-05	1.36
16726224	STIP1	NM_001282652	1.21E-04	1.17
16683644	SYF2	NM_015484	1.53E-04	-1.19
16699590	TAF1A	NM_001201536	5.73E-05	1.40
16677304	TATDN3	NM_001042552	1.61E-04	-1.32
16943284	TBC1D23	NM_001199198	2.46E-04	1.07
16898872	TEX261	NM_144582	1.77E-04	1.27
16701407	TFB2M	NM_022366	1.51E-04	1.21
16776905	TFDP1	NR_026580	7.30E-05	1.32
17059828	TFPI2	NM_001271003	5.00E-05	1.50
16963241	TFRC	NM_001128148	6.15E-05	2.25
17069045	TGS1	NM_024831	1.09E-05	1.20
16799315	THBS1	NM_003246	1.43E-04	1.62
16804631	TICRR	NM_152259	1.70E-04	1.28
17088527	TLR4	NM_003266	3.69E-05	1.25
17009620	TMEM14A	NM_014051	2.34E-04	1.31

16917114	TMX4	ENST00000246024	8.49E-05	1.40
16846734	TOB1	NM_001243877	1.03E-04	-1.22
16935427	TOB2	NM_016272	6.38E-05	-1.16
16930093	TOMM22	NM_020243	7.51E-05	1.24
16737543	TP53I11	NM_001258320	2.14E-04	-1.39
16859253	TPM4	NM_001145160	4.78E-05	-1.15
16732088	TRAPPC4	ENST00000359005	2.43E-05	1.30
16943404	TRMT10C	NM_017819	1.97E-04	1.29
16917030	TRMT6	NM_015939	2.09E-04	1.27
17080342	TRPS1	NM_001282903	1.06E-04	-1.23
16831388	TTC19	NM_001271420	1.88E-07	-1.25
17097052	TXN	NM_003329	1.61E-04	1.21
16865737	U2AF2	NM_001012478	5.82E-05	1.23
16731822	UBE4A	NM_001204077	5.29E-05	-1.21
16807996	UBR1	NM_174916	1.54E-04	-1.19
16953454	UCN2	NM_033199	1.86E-04	-1.57
16932300	UFD1L	NM_001035247	5.88E-06	1.48
16857258	UHRF1	NM_001048201	1.86E-05	1.47
16760257	VWF	NM_000552	6.95E-05	-1.22
16974280	WDR1	NM_005112	1.89E-04	1.15
16861318	WDR62	NM_001083961	3.15E-05	1.27
17045656	YKT6	NM_006555	1.39E-05	1.22
17084936	ZCCHC7	NM_001289119	1.99E-04	-1.27
17098900	ZDHHC12	NM_032799	1.49E-04	1.23
16861997	ZFP36	NM_003407	2.44E-04	-1.46
16706463	ZMIZ1	NM_020338	5.81E-05	1.13
17050361	ZNF277	NM_021994	9.08E-07	-1.32
16939934	ZNF35	NM_003420	2.35E-04	1.20

Supplementary Table 1. Differentially expressed transcripts resulting from the comparison between overexpressing miR-205 cells and controls in A375 cells derived from an analysis of variance (FDR <0.05). Out of 243 transcripts, 152 were up-regulated (positive fold change) and 91 were downregulated (negative fold change).
